# Multiple loss-of-function mutations of carotenoid cleavage dioxygenase 4 reveal its major role in both carotenoid level and apocarotenoid composition in flue-cured mature tobacco leaves

**DOI:** 10.1038/s41598-023-39692-4

**Published:** 2023-08-10

**Authors:** Hiroshi Magome, Masao Arai, Kiyoshi Oyama, Ryo Nishiguchi, Yoshimitsu Takakura

**Affiliations:** Leaf Tobacco Research Center, Japan Tobacco, Inc., 1900 Idei, Oyama, Tochigi 323-0808 Japan

**Keywords:** Plant breeding, Plant physiology, Secondary metabolism

## Abstract

Apocarotenoid volatiles contribute to the flavor of many agricultural products. In many flowering plants, carotenoid cleavage dioxygenase 4 (CCD4) is involved in the decomposition of carotenoids and resultant production of C13-apocarotenoids, such as β-ionone derived from β-carotene. To understand the possible role of tobacco CCD4 genes (*NtCCD4-S*, *NtCCD4-T1* and *NtCCD4-T2*) in these processes, we analyzed loss-of-function phenotypes. RNA interference transgenic plants showed yellow color in mature (senescent) leaves. Mature leaves of chemically induced double-mutant plants showed a stronger yellow color, and those of triple-mutant plants showed a pronounced yellow color. Carotenoid analysis of the leaves from mutants showed that lutein and β-carotene increased in line with the degree of color change compared to wild type, whereas there was little change in green color in their young leaves. This result indicates that CCD4s are important for the decomposition of carotenoids in the tobacco leaf maturation process. Analysis of apocarotenoids in flue-cured leaves of the multiple-mutant plants showed that many compounds, including megastigmatrienones, were decreased in comparison to wild type, whereas intriguingly β-ionone and dihydroactinidiolide were increased. Our results suggest that CCD4s play a key role in both carotenoid level and apocarotenoid composition in flue-cured mature tobacco leaves.

## Introduction

Carotenoids, formed from C40 isoprenoids, are pigments most of which representing red, orange or yellow color. In plants, they play important roles in photosynthesis together with chlorophylls, including a photoprotective role under excess light, and function as antioxidants against oxidative stress^1,2^. Yellowing of a senescent leaf occurs when carotenoids become dominant because their breakdown is slower than that of chlorophylls. β-Carotene and lutein are major carotenoids in leaf and are biosynthesized from geranylgeranyl diphosphate via a multistep process in plastids, including chloroplasts^1,3^. The endogenous level of carotenoids is regulated by a balance of their biosynthesis and decomposition; the latter consists of enzymatic and non-enzymatic processes that produce various breakdown products called apocarotenoids^4^.

Tobacco (*Nicotiana tabacum* L.) is a major commercial non-food crop; its cured (dried) leaves are used for tobacco products, such as cigarettes, cigars and heated tobacco products. Because the flavor imparted upon smoking is important, the flavor-related constituents are of interest to the tobacco industry and have been studied for several decades^5–9^. It is widely known that compounds derived from carotenoids, called apocarotenoids, contribute flavor to tobacco products, and tobacco is one of the richest sources of apocarotenoids, with almost 100 compounds being identified^10^. Decrease in carotenoids in tobacco leaf during maturing (senescence) and curing (drying) has been reported^11^. Recently, proteomic analysis of tobacco leaf during flue-curing showed the possible involvement of lipoxygenases and peroxidases in carotenoid metabolism^12^. However, no enzyme involved in the conversion of carotenoids into apocarotenoids in tobacco leaf has been identified thus far.

Apocarotenoids are produced in flowering plants when carotenoids decompose upon catalysis by carotenoid cleavage dioxygenase (CCD), which cleaves their conjugated double bonds. In the tobacco allotetraploid genome, 19 genes encoding CCD have recently been identified; on the basis of the deduced amino acid sequence similarity to those of other flowering plants, they were assigned to 9 clades: CCD1, -4, -7, -8 and -L/ZAS, and 9-*cis*-epoxycarotenoid cleavage dioxygenases 2, -3, -5 and -6^13^. Their substrate specificities and physiological roles are variable in many plant species, ranging from the biosynthesis of phytohormone abscisic acid by 9-*cis*-epoxycarotenoid cleavage dioxygenases^14,15^ or that of strigolactone by CCD7 and -8^16^ to the production of zaxinone, a signal molecule in rice, by CCD-L/ZAS^17^. Functional analysis of CCD1 and CCD4 revealed that they are involved in flavor production via carotenoid decomposition^18^. CCD4 also influences carotenoid accumulation in various tissues; for example, a loss-of-function of CCD4 caused by RNA interference (RNAi) in a white-flower variety of chrysanthemum results in increased levels of carotenoids and in yellow flowers^19^. Similarly, CCD4-RNAi potato has yellower tubers and flowers with increased carotenoids^20^. The dry seeds of an Arabidopsis *ccd4* mutant accumulate carotenoids above the level found in wild type^21^.

Here, we report the carotenoid and apocarotenoid profiles of tobacco plants with multiple mutations of the CCD4 genes *NtCCD4-S*, *NtCCD4-T1* and *NtCCD4-T2*. Our results suggest that NtCCD4s play a key role in both carotenoid level and apocarotenoid composition in flue-cured mature tobacco leaves.

## Results

### RNA interference (RNAi)-mediated silencing of *NtCCD4* results in yellow mature leaves due to the accumulation of carotenoids

**Figure 1:**
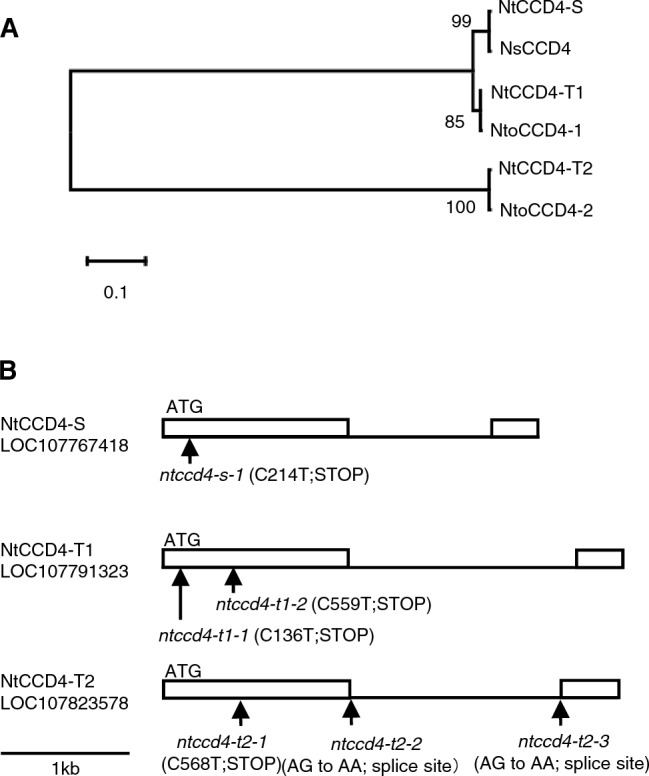
*Nicotiana tabacum* carotenoid cleavage dioxygenase 4 (CCD4). (**A**) Phylogenetic tree of the CCD4 amino acid sequences in *N. tabacum* (Nt), *N. sylvestris* (Ns) and *N. tomentosiformis* (Nto) with bootstrap values. (**B**) Physical map of mutations of *NtCCD4-S1* (LOC107767418), *NtCCD4-T1* (LOC107791323) and *NtCCD4-T2* genes (LOC107823578). Numbers denote distance (in nucleotides) from the initiation codon. White boxes, horizonal bars, and arrows show exons, introns and the location of mutations, respectively.

**Figure 2:**
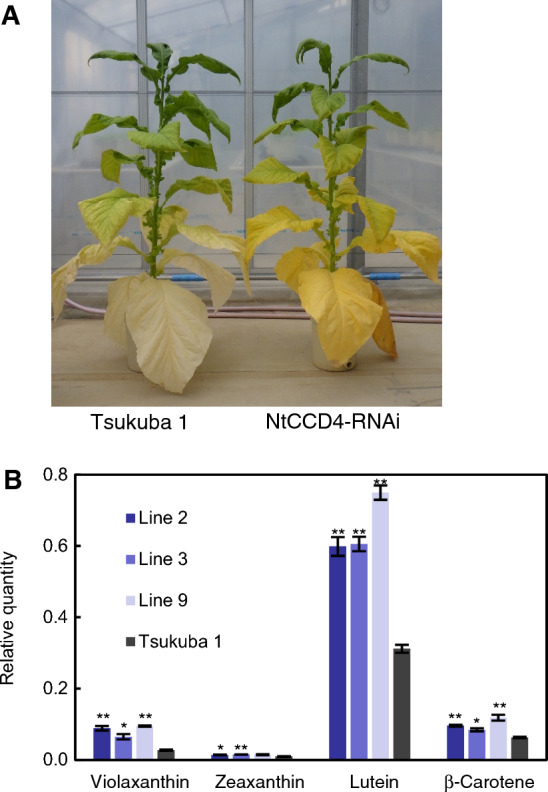
Phenotypes of the control cultivar Tsukuba 1 and RNA interference (RNAi) tobacco plants. (**A**) Twenty-two-week-old plants of Tsukuba 1 and Line 3 of *N. tabacum* (Nt) carotenoid cleavage dioxygenase 4 (CCD4) RNAi. (**B**) Carotenoid levels in mature leaves of NtCCD4-RNAi (Lines 2, 3 and 9) and Tsukuba 1. Data are the mean of three biological replicates ± SEM. Significant differences from Tsukuba 1 (Student’s *t*-test): **p < 0.01; *p < 0.05.

Allotetraploid tobacco (*N. tabacum*) has three CCD4 genes (*NtCCD4a*, -*b* and -*c*) that are likely derived from the diploid ancestors *Nicotiana sylvestris* and *Nicotiana tomentosiformis*^13^. Phylogenetic relationship of CCD4s between *N. tabacum* and the two ancestors is shown in Fig. 1A. The coding region of *NtCCD4c* is 100% identical to the nucleotide sequence of *N. sylvestris* CCD4 protein and therefore is designated hereafter as *NtCCD4-S*. Likewise, *NtCCD4b* and *-a*, which are 100% and 99% identical to *N. tomentosiformis* CCD4-1 and CCD4-2 sequences respectively, are designated as *NtCCD4-T1* and *NtCCD4-T2* in the present study. The three *N. tabacum* CCD4s are over 95% identical to each other at the amino acid sequence level (Supplementary Table S1). To deduce the physiological role of CCD4s in *N. tabacum*, we generated CCD4-RNAi transgenic plants (cultivar Tsukuba 1) designed to silence all three NtCCD4 genes. Three independent lines (2, 3 and 9) with less than 5% remaining mRNA levels of *NtCCD4-T1* were selected (Supplementary Fig. S1) and grown in a greenhouse. The phenotypes of the *N. tabacum* CCD4-RNAi plants were morphologically similar to non-transformed plants but their mature (senescent) leaves were yellow (Fig. 2A). We analyzed the endogenous carotenoid levels in the mature leaves of the plants and compared them with those of the non-transformed Tsukuba 1 plants. Semi-quantitative analysis showed that the levels of all four carotenoids, lutein (1.9–2.4 fold), β-carotene (1.3–1.9 fold), violaxanthin (2.4–3.5 fold) and zeaxanthin (1.5–1.6 fold), were increased in the mature leaves of NtCCD4-RNAi plants (Fig. 2B). These results suggest that silencing of *NtCCD4* genes results in yellow mature leaves because of the accumulation of carotenoids.

### *NtCCD4*-triple and -double mutant plants produced yellow mature leaves

**Figure 3:**
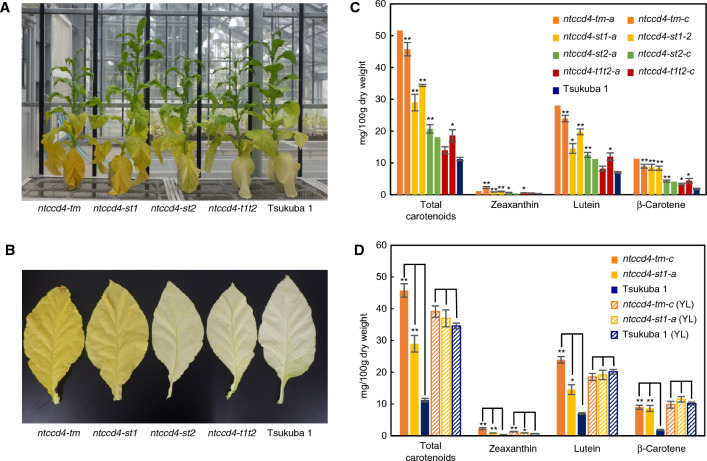
Phenotypes of *N. tabacum* (Nt) plants with multiple mutations of carotenoid cleavage dioxygenase 4 (CCD4) grown in a greenhouse. (**A**) Thirteen-week-old plants of the triple mutant (*tm*), double mutants (*st1*, *st2* and *t1t2*) and Tsukuba 1. (**B**) Color phenotype of mature leaves from the CCD4 multiple-mutant plants. (**C**) Carotenoid levels in the mature leaves of two triple-mutant lines (dark orange), double-mutant lines (light orange, green and red), and control cultivar (blue). Data are the mean of three biological replicates ± SEM, except for *ntccd4-tm-a* (*n* = 1) and *ntccd4-st2-c* (*n* = 2). Significant differences from Tsukuba 1 (Student’s *t*-test): **p < 0.01; *p < 0.05. (**D**) Comparison of carotenoid levels between mature (from the lower stalk) and young (from the upper stalk) leaves. The endogenous carotenoid levels in the mature leaves of each strain were compared with those in the young leaves (YL). Data are the mean of three biological replicates ± SEM. Significant differences from Tsukuba 1 (Student’s *t*-test): **p < 0.01; *p < 0.05.

To clarify the effect of loss-of-function mutations of *NtCCD4* genes on tobacco leaf, we screened genotypes homozygous for the following gene mutations: one for *NtCCD4-S* (*ntccd4*-*s-1*), two for *NtCCD4-T1* (*ntccd4*-*t1-1* and *ntccd4*-*t1-2*) and three for *NtCCD4-T2* (*ntccd4*-*t2-1, ntccd4-t2-2* and *ntccd4-t2-3*) from our ethyl methanesulfonate—generated mutant lines of Tsukuba 1^22,23^ (Fig. 1B). The mutations in *ntccd4-s-1*, *ntccd4-t1-1*, *ntccd4-t1-2* and *ntccd4-t2-1* are substitutions of C to T in each exon 1, which results in formation of the stop codon. Those in *ntccd4-t2-2* and *ntccd4-t2-3* are substitutions of G to A at 5ʹ and 3ʹ ends of the splice site in the intron, respectively, each resulting in a premature stop codon (Fig. 1B). Two double-mutant lines for *NtCCD4-S* and *-T1* genes (*ntccd4-st1-1* and *ntccd4-st1-2*) were selected by genotyping F_2_ lines derived from F_1_ lines that had been generated by crossing between single mutants (*ntccd4-s-1* and *ntccd4-t1-1* for *ntccd4-st1-1* and *ntccd4-s-1* and *ntccd4-t1-2* for *ntccd4-st1-2*). In the homozygous mutants, the colors of mature leaves in the lower positions of the *ntccd4-st1* double-mutant plants were yellower than any of those in single mutants or Tsukuba 1 (Supplementary Fig. S2), as was the case with the RNAi transgenic plants. We analyzed endogenous carotenoid profiles of mature leaves in the double mutants (*ntccd4-st1-1* and *ntccd4-st1-2*) and compared them with those of segregated wild-type plants without any mutations in *NtCCD4*s (sibling lines of *ntccd4-st1-1* and *ntccd4-st1-2*, referred to as WT1 and WT2, respectively) (Supplementary Fig. S3). As with the RNAi transgenic plants, we found increased levels of carotenoids in the mature leaves of both double-mutant lines. These results indicate that these phenotypes of the double-mutant plants are caused by the loss of function of both *NtCCD4-S* and *-T1* genes. To obtain the triple mutant and other genotypes of double mutants, we next crossed the *ntccd4-st1-2* double mutant with the *ntccd4-t2-3* single mutant. The resulting progeny, two *ntccd4*-*st1t2* triple-mutant lines (*ntccd4-tm-a* and *ntccd4-tm-c*), a *ntccd4-st1* double-mutant line (*ntccd4-st1a*), two *ntccd4-st2* double-mutant lines (*ntccd4-st2-a* and *ntccd4-st2c*) and two *ntccd4-t1t2* double mutants (*ntccd4-t1t2-a* and *ntccd4-t1t2-c*), were isolated and together with the *ntccd4-st1-2* double mutant (as the parent line) were used for further analysis. We observed the leaf color of the multiple-mutant plants grown in a greenhouse (Fig. 3). Compared to the control cultivar Tsukuba 1, the triple mutant phenotype showed a pronounced yellow color in the mature leaves, followed by the *ntccd4-st1* double mutant and then two other double mutants, *ntccd4-st2* and *ntccd4-t1t2*. We also found some individuals among the multiple-mutant lines with various signs of growth retardation that was not likely related to the CCD4 genotype but due to unidentified background mutations from single mutants, because the RNAi transgenic plants were morphologically normal (Fig. 2A). We further analyzed the adaxial color of mature leaves using the CIEL*a*b* system (Table 1). Compared to the control, especially a* (positive value for redness) and b* (positive value for yellowness) values of the triple mutants were most increased, followed by those of *ntccd4-st1* and then *ntccd4-st2 and ntccd4-t1t2* (Table 1). These results were consistent with observations by the naked eye (Fig. 3B).

**Table 1:** Color of mature leaves of *N. tabacum* (Nt) plants with multiple mutations of carotenoid cleavage dioxygenase 4 (CCD4) grown in a greenhouse.

Lines	CCD4 genotype	L*a*b* color values		
		L*	a*	b*
*ntccd4-tm-a*	Triple mutant	76.35 ± 0.20^a^	19.02 ± 0.34^a^	64.43 ± 0.38^a^
*ntccd4-tm-c*	Triple mutant	76.15 ± 0.20^a^	19.51 ± 0.28^a^	63.55 ± 0.42^a^
*ntccd4-st1-a*	Double mutant	78.66 ± 0.20^b^	13.97 ± 0.26^b^	59.52 ± 0.53^b^
*ntccd4-st1-2*	Double mutant	78.42 ± 0.13^b^	14.78 ± 0.14^b^	60.57 ± 0.41^b^
*ntccd4-st2-a*	Double mutant	81.96 ± 0.30^c^	6.52 ± 0.38^c^	43.35 ± 1.31^c^
*ntccd4-st2-c*	Double mutant	83.31 ± 0.12^c^	4.20 ± 0.12^d^	37.62 ± 0.35^c^
*ntccd4-t1t2-a*	Double mutant	82.27 ± 0.31^c^	4.78 ± 0.25^d^	37.61 ± 0.70^c^
*ntccd4-t1t2-c*	Double mutant	84.57 ± 0.17^d^	2.72 ± 0.13^e^	33.71 ± 0.84^d^
Tsukuba1	Wild type	84.18 ± 0.14^d^	2.32 ± 0.14^e^	33.62 ± 0.59^d^

The adaxial leaf color of L*, a* and b* values are shown. Data are the mean of 18 points in three leaves (six points per leaf) ± SEM. Letters indicate statistically significant differences between samples by Steel–Dwass test (*p* < 0.05).

### *NtCCD4* triple- and double-mutant plants highly accumulate carotenoids in their mature leaves

We next analyzed endogenous carotenoid profiles of these *ntccd4* multiple-mutant lines. The levels of total carotenoids and of lutein, β-carotene and zeaxanthin individually were higher in mature leaves of all the *ntccd4* multiple-mutant lines than in those of Tsukuba 1. The triple-mutant lines showed the highest accumulation (total carotenoids, 4.1–4.6 fold; lutein, 3.4–4.0 fold; β-carotene, 4.9–6.2 fold and zeaxanthin, 3.3–6.8 fold), and to a lesser extent the *ntccd4-st1* double mutant, and then the other double-mutant lines (Fig. 3C). The minor carotenoids α-carotene and lycopene were not detected in any mutant lines nor in Tsukuba 1 (data not shown). We further analyzed the influence of *NtCCD4* mutations in young leaves with green color. Carotenoid levels of the upper green leaves of the triple mutant *ntccd4-tm-c*, the double mutant *ntccd4-st1-a* and Tsukuba 1 were compared with those of mature leaves (Fig. 3D). Interestingly, carotenoid levels of the green leaves of each genotype were similar to those of Tsukuba 1, indicating that the influence of *NtCCD4* mutations in green leaves is small. Levels of total carotenoids, zeaxanthin and lutein in mature leaves of the triple mutants were higher than those in young leaves of Tsukuba 1, indicating that decomposition of carotenoids during senescence is mostly blocked in the triple mutants. Taken together, these results indicate that CCD4s in *N. tabacum* are key proteins for decomposition of carotenoids in leaf maturation.

### Flue-cured leaves of field-grown *NtCCD4* multiple mutants showed drastic changes in their apocarotenoid profiles

**Figure 4:**
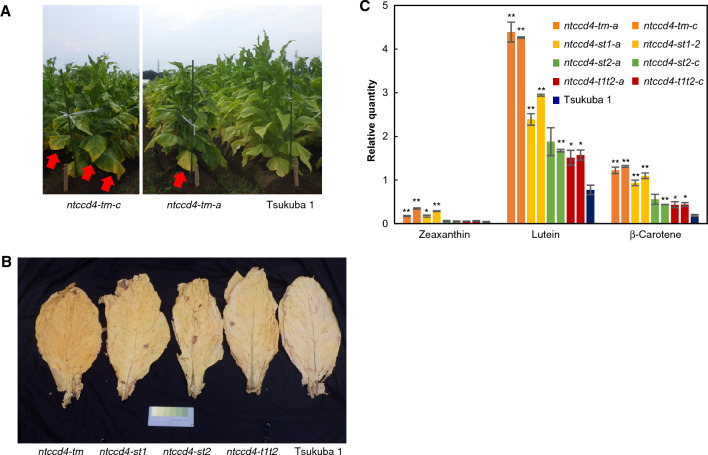
Phenotypes of *N. tabacum* (Nt) plants with multiple mutations of carotenoid cleavage dioxygenase 4 (CCD4) grown in the field. (**A**) Thirteen-week-old triple-mutant (*tm*) strains (*a* and *c*) and Tsukuba 1 are shown. Note that the yellow color shown by arrows was seen in the lower stalk of the triple mutant leaves. (**B**) Colors of the flue-cured leaves of ntccd4 multiple-mutant plants. Flue-cured leaves taken from the lower part of the stalk of a triple mutant (*tm*) and double mutants (*st1*, *st2* and *t1t2*) are shown next to the control cultivar, Tsukuba 1. (**C**) Carotenoid levels in the flue-cured leaves of *N. tabacum* (Nt) plants with multiple mutations of carotenoid cleavage dioxygenase 4 (CCD4). Data are the mean of three biological replicates ± SEM. Significant differences from Tsukuba 1 (Student’s *t*-test): **p < 0.01; *p < 0.05.

Given that enzymatic decomposition of carotenoids is blocked in *ntccd4* multiple mutants, the character of their flue-cured leaves may also be affected. To clarify this, we grew them and Tsukuba 1 in the field and analyzed their flue-cured leaves. We could distinguish the yellow mature leaves of the triple mutants (Fig. 4A) and the double mutant *ntccd4-st1* (data not shown) from mature leaves of Tsukuba 1 in the field but could not do so for those of other double mutants (data not shown), most likely because of their weak phenotypes*.* Leaves at the lower part of the stalk of all strains (including Tsukuba 1) were harvested, and then the leaves were flue-cured according to the local production protocol of Tsukuba 1 (Supplementary Table S2). We found that the yellow color of *ntccd4* multiple mutants was retained after flue-curing; compared to Tsukuba 1, the yellow color was strongest in the flue-cured leaves of the triple mutants, followed by those of the *ccd4-st1* double mutant and then those of the other double mutants, *ntccd4-st2 and ntccd4-t1t2* (Fig. 4B). This was confirmed by measuring adaxial leaf color using the CIEL*a*b* system. We found that compared to Tsukuba 1, especially a* (positive value for redness) and b* (positive value for yellowness) values of the triple mutants were most increased, followed by those of *ntccd4-st1* and then *ntccd4-st2* and *ntccd4-t1t2* (Table 2).

**Table 2:** Color of flue-cured leaves from *N. tabacum* (Nt) plants with multiple mutations of carotenoid cleavage dioxygenase 4 (CCD4) grown in the field.

Lines	CCD4 genotype	L*a*b* color values		
		L*	a*	b*
*ntccd4-tm-a*	Triple mutant	68.10 ± 0.32^b^	18.02 ± 0.23^b^	50.58 ± 0.50^b^
*ntccd4-tm-c*	Triple mutant	65.83 ± 0.42^a^	20.26 ± 0.38^a^	53.36 ± 0.46^a^
*ntccd4-st1-a*	Double mutant	69.03 ± 0.32^bcd^	16.66 ± 0.29^cd^	50.14 ± 0.48^b^
*ntccd4-st1-2*	Double mutant	68.82 ± 0.27^bcd^	17.18 ± 0.39^bc^	51.09 ± 0.72^ab^
*ntccd4-st2-a*	Double mutant	68.21 ± 0.37^bcd^	14.51 ± 0.26^df^	47.75 ± 0.84^bc^
*ntccd4-st2-c*	Double mutant	68.22 ± 0.33^bd^	15.47 ± 0.35^de^	47.37 ± 0.93^bc^
*ntccd4-t1t2-a*	Double mutant	69.22 ± 0.32^bd^	14.79 ± 0.2^e^	48.16 ± 0.73^b^
*ntccd4-t1t2-c*	Double mutant	69.83 ± 0.39^bd^	13.30 ± 0.23^f^	44.42 ± 0.61^c^
Tsukuba1	Wild type	70.54 ± 0.54^d^	11.13 ± 0.21^g^	38.64 ± 0.78^d^

The adaxial leaf color of L*, a* and b* values are shown. Data are the mean of 24 points in four leaves (six points per leaf from each individual test area) ± SEM. Letters indicate statistically significant differences between samples by Steel–Dwass test (*p* < 0.05).

We next analyzed the endogenous carotenoid and apocarotenoid profiles of these flue-cured leaves. Semi-quantitative carotenoid analysis showed that the levels of lutein, β-carotene and zeaxanthin were increased in all the *ntccd4* multiple-mutant lines compared with those of Tsukuba 1; the triple-mutant lines showed the highest accumulation (lutein, 5.5–5.7 fold; β-carotene, 6.6–7.0 fold; zeaxanthin, 4.8–9.6 fold) and to a lesser extent the double mutant *ntccd4-st1s* and then *ntccd4-st2s and ntccd4-t1t2s* (Fig. 4C), as was the case with the uncured mature leaf samples grown in a greenhouse (Fig. 3C). Semi-quantitative apocarotenoid analysis detected 16 compounds from these flue-cured leaves, of which 12 compounds tended to decrease compared to Tsukuba 1, including four isomers of megastigmatrienones (0.1–0.2 fold; numbers in parentheses show hold change in the triple mutant lines), β-damascenone (0.3–0.4 fold), β-damascone (0.1 fold), Blumenol A (0.3–0.5 fold) and Blumenol C (0.1–0.3 fold) and 3-oxo-α-ionol (0.2–0.3 fold) (Table 3). The decrease was seen in all the *ntccd4* multiple-mutant lines, but especially in the triple-mutant lines. The compound 3-hydroxy-7,8-dehydro- β-ionol was tended to decrease in the multiple-mutant lines but, as an exception, that of the triple mutant *ntccd4-tm-a* was unchanged. Intriguingly, in the multiple mutants two compounds were increased, β-ionone (3.5–3.6 fold) and dihydroactinidiolide (4.8–5.4 fold). There was also a trend for β-cyclocitral to increase (1.6–1.7 fold) in the multiple mutants.

**Table 3:** Apocarotenoid profiles of flue-cured leaves from N. tabacum (Nt) plants with multiple mutations of carotenoid cleavage dioxygenase 4 (CCD4) grown in the field.

Compound	Triple mutant		Double mutant						Wild type
	*ntccd4-tm-a*	*ntccd4-tm-c*	*ntccd4-st1-a*	*ntccd4-st1-2*	*ntccd4-st2-a*	*ntccd4-st2-c*	*ntccd4-t1t2-a*	*ntccd4-t1t2-c*	Tsukuba1
Dihydroactinidiolide	0.1018 ± 0.0104**	0.0905 ± 0.0024**	0.0476 ± 0.0013**	0.0506 ± 0.0016**	0.0302 ± 0.0015*	0.0258 ± 0.0007	0.0269 ± 0.0012*	0.0294 ± 0.0009*	0.0189 ± 0.0020
β-ionone	0.0343 ± 0.0029**	0.0334 ± 0.0007**	0.0220 ± 0.0005**	0.0277 ± 0.0007**	0.0117 ± 0.0005	0.0117 ± 0.0007	0.0119 ± 0.0007	0.0135 ± 0.0001**	0.0096 ± 0.0006
β -damascenone	0.0172 ± 0.0019*	0.0133 ± 0.0005**	0.0171 ± 0.0004**	0.0183 ± 0.0013*	0.0286 ± 0.0024	0.0297 ± 0.0027	0.0301 ± 0.002	0.0302 ± 0.0009	0.0442 ± 0.0047
Megastigmatrienone(1)/MEG5	0.0280 ± 0.0011**	0.0339 ± 0.0013**	0.1325 ± 0.0067**	0.1473 ± 0.0108**	0.1608 ± 0.0131**	0.1701 ± 0.0049**	0.1715 ± 0.0139*	0.1879 ± 0.0005**	0.2817 ± 0.0146
Megastigmatrienone(2)/MEG1	0.0512 ± 0.0025**	0.0637 ± 0.0031**	0.3502 ± 0.0166**	0.4151 ± 0.0331**	0.4505 ± 0.0365**	0.4790 ± 0.0140**	0.4830 ± 0.0398*	0.5436 ± 0.0024**	0.8068 ± 0.0423
Megastigmatrienone(3)/MEG4	0.0831 ± 0.0030**	0.0965 ± 0.0045**	0.5042 ± 0.0235**	0.5683 ± 0.0460**	0.6209 ± 0.0478**	0.6541 ± 0.0142**	0.6754 ± 0.0552*	0.7607 ± 0.0059**	1.0952 ± 0.0589
Megastigmatrienone(4)/MEG3	0.0327 ± 0.0012**	0.0386 ± 0.0017**	0.1785 ± 0.0087**	0.1962 ± 0.0161**	0.2234 ± 0.0169**	0.2341 ± 0.0056**	0.2382 ± 0.019*	0.2651 ± 0.0016**	0.3818 ± 0.0205
3-hydroxy- β -damascone	0.0726 ± 0.0038**	0.0730 ± 0.0027**	0.1863 ± 0.0083**	0.1878 ± 0.0164**	0.2645 ± 0.0198*	0.2855 ± 0.0134*	0.2756 ± 0.0186*	0.3007 ± 0.0014*	0.4185 ± 0.0226
3-hydroxy-5,6-epoxy- β -ionol	0.0582 ± 0.0093*	0.0351 ± 0.0023**	0.0458 ± 0.0079**	0.0479 ± 0.0036**	0.0595 ± 0.0045*	0.0640 ± 0.0064*	0.0684 ± 0.0054*	0.0777 ± 0.0038*	0.1616 ± 0.0189
4-(3-hydroxybuthylidene)-3,5,5-trimethyl-2-cyclohexen-1-one	0.0085 ± 0.0006*	0.0057 ± 0.0006*	0.0152 ± 0.0028	0.0131 ± 0.0008	0.0095 ± 0.0008	0.0105 ± 0.0008	0.0114 ± 0.0007	0.0112 ± 0.0006	0.0210 ± 0.0033
β -cyclocitral	0.0056 ± 0.0005*	0.0058 ± 0.0002**	0.0061 ± 0.0009	0.0071 ± 0.0001**	0.0058 ± 0.0008	0.0049 ± 0.0002*	0.0042 ± 0.0004	0.0056 ± 0.0001**	0.0035 ± 0.0003
3-hydroxy-7,8-dehydro- β -ionol	0.0064 ± 0.0015	0.0025 ± 0.0001**	0.0023 ± 0.0004**	0.0024 ± 0.0003**	0.0037 ± 0.0001*	0.0046 ± 0.0004	0.0050 ± 0.0002	0.0035 ± 0.0003*	0.0063 ± 0.0005
Blumenol A	0.0857 ± 0.0120	0.0533 ± 0.0031*	0.0805 ± 0.0039*	0.0871 ± 0.0081	0.1166 ± 0.0138	0.1000 ± 0.0075	0.0952 ± 0.0061	0.1225 ± 0.0128	0.1563 ± 0.0195
Blumenol C	0.0044 ± 0.0005**	0.0024 ± 0.0003**	0.0059 ± 0.0005**	0.0112 ± 0.0004	0.0059 ± 0.0001**	0.0071 ± 0.0004**	0.0099 ± 0.0007*	0.0103 ± 0.0002	0.0162 ± 0.0015
3-oxo- α -ionol	0.1383 ± 0.0207*	0.0735 ± 0.0036**	0.2220 ± 0.0479**	0.2099 ± 0.0110*	0.1418 ± 0.0166*	0.1615 ± 0.0247*	0.1938 ± 0.0209*	0.1886 ± 0.0072*	0.4194 ± 0.0595
β -damascone	0.0007 ± 0.0001**	0.0009 ± 0.0001**	0.0090 ± 0.0003**	0.0124 ± 0.0010	0.0103 ± 0.0003*	0.0093 ± 0.0006*	0.0088 ± 0.0002**	0.0131 ± 0.0010	0.0131 ± 0.0005

Data are the mean of three biological replicates ± SEM. Increases compared to Tsukuba 1 are indicated in bold. Significant differences from the wild type (Student’s t-test): **p < 0.01; *p < 0.05.

## Discussion

Decomposition of carotenoids and the resultant production of apocarotenoids in mature tobacco leaf have been reported thoroughly, but genes involved in this process have not been clarified. Here, by using a reverse genetic approach, we demonstrated that the CCD4s play a predominant role in this process in mature tobacco leaves.

Carotenoid analyses showed that the triple mutants and to a lesser extent double mutants accumulated carotenoids in mature leaves (Fig. 3C). This result suggests that *N. tabacum* CCD4s share a common role in the downregulation of carotenoid levels in leaves. Although previous proteomic analysis of tobacco leaf during flue-curing suggested the involvement of lipoxygenases and peroxidases in carotenoid metabolism^12^, the similarity in the levels of carotenoid increase in the triple mutants in the mature (Fig. 3C) and flue-cured leaves (Fig. 4C) in the present study also suggests a key role of *N. tabacum* CCD4s after harvest in flue-curing. In CCD4-RNAi potato, which is another solanaceous plant, the effect of *CCD4* knockdown on carotenoid level was clear in tubers and flower petals, but not in leaves^20^. The reason why the double-mutant phenotype of *ntccd4-st1* was stronger than those of *ntccd4-st2* and *ntccd4-t1t2* in both leaf color and carotenoid level is not currently clear. A simple explanation is that the ratio of contribution by the genes is uneven; the role of *NtCCD4-T2* gene might be relatively lower than those of other genes in carotenoid metabolism. To clarify this, further analysis of new sets of double mutants made from distinct single-mutant alleles is required.

In the multiple mutants, the high-carotenoid phenotype was observed in the mature leaves, but there was little change in their young green leaves (Fig. 3D), indicating that the influence of *NtCCD4* mutations in young leaves is much smaller than that in mature leaves. However, *NtCCD4* genes are expressed in all leaves at the flowering stage^13^, suggesting that these genes are ubiquitously expressed in leaf. These facts may support a mechanism hypothesized by Rottet et al*.*^24^ that carotenoids are sequestered away from CCD4 in young leaves. In Arabidopsis, CCD4 localizes in plastoglobules, which are thylakoid-associated lipoprotein particles^24^. The authors proposed that carotenoids are associated with the light-harvesting complex proteins in the thylakoids of chloroplasts, thus making the carotenoids inaccessible to CCD4 in the plastoglobules. When light-harvesting complex proteins are degraded during senescence, the carotenoids in the plastoglobules become accessible to CCD4-dependent decomposition^24^. If this hypothesis is true for tobacco plants, this may explain the discrepancy between the young leaves and the mature leaves in the present study.

Apocarotenoid analysis of the flue-cured leaves showed that 12 out of 16 compounds, including megastigmatrienones, were decreased, whereas β-ionone and dihydroactinidiolide were increased in the *ntccd4* multiple-mutant plants (Table 3). It should be noted that these apocarotenoids are not only present as free forms but also as glycoside forms in tobacco leaves^9,25^ and also in Arabidopsis leaves^26^. Similarly, in our preliminary analysis, the signal strength of apocarotenoids was elevated in leaf extraction by the highly polar solvent used in this study (methanol) more than they were by a low-polar solvent (hexane; data not shown), suggesting our data was likely to be the total of the free and glycosylated forms of apocarotenoid. In Arabidopsis, four major apocarotenoid glycosides are decreased in leaves of the *ccd4-1* mutant plants^26^. This result is consistent with our result that the majority of apocarotenoids are decreased in the flue-cured leaves of the *ntccd4* multiple mutants, and supports the idea that CCD4s play a key role in apocarotenoid production in tobacco leaves. However, the increased levels of β-ionone and dihydroactinidiolide seem to be paradoxical, because it has been reported that canonical CCD4 protein cleaves carotenoids at the 9, 10 or 9ʹ, 10′ double bond to generate β-ionone and other C13-apocarotenoids in several flowering plants^27^, and the primary structure of *N. tabacum* CCD4s is closely related to those of canonical Arabidopsis and potato CCD4s (Supplementary Table S1). One possible reason for this paradoxical result is the likely presence of other enzymes including other CCDs. Another possible reason is the involvement of a non-enzymatic process. Previously, it was reported that β-ionone, dihydroactinidiolide and β-cyclocitral are produced from β-carotene by in vitro ^1^O_2_ oxidation, and they are also produced in Arabidopsis leaves subjected to high-light stress under which ^1^O_2_ is produced^28^. To clarify the expression mechanism of apocarotenoid profiles in the *ntccd4* multiple mutants, a functional analysis of CCD4 proteins and temporal monitoring of apocarotenoids in tobacco leaves during harvest and flue-curing would be needed.

The *ntccd4* multiple-mutant plants generated in this study show promise for improving tobacco leaf quality. Their flue-cured leaves contain increased levels of β-ionone and dihydroactinidiolide (Table 3) that are used as fragrances. The accumulated carotenoids (Fig. 4C) also could be a source of flavor by additional processes such as thermal decomposition or oxidation. The characteristics of the double mutant *ntccd4-st1* were weaker than those of the triple mutants, but the former might be more useful than the latter because the decrease in levels of other apocarotenoids, such as megastigmatrienones, which are thought to contribute to tobacco flavor^29^, was milder (Table 3), and in terms of breeding, introgression of two rather than three loci into another cultivar is achieved more easily.

## Materials and methods

### Plant materials and growth conditions

A flue-cured tobacco (*N. tabacum)* cultivar Tsukuba 1 was used in this study. In the greenhouse, plants were grown in pots at 23–25 °C under natural day-length conditions. Topping was done at the early blossom stage. At harvest, mature leaves from the lower stalk (two quarters from the bottom) and young leaves from the upper stalk (the remaining above three-quarters) were collected from 13-week-old plants and then stored at − 80 °C. In the field experiment, plants were grown in an experimental field at Oyama City, Tochigi Prefecture, Japan in 2020. The topping was done at the early blossom stage. Mature leaves at the lower stalk (two quarters from the bottom) of 14-week-old plants were harvested and then flue-cured on the basis of the local production protocol of Tsukuba 1 (Supplementary Table S2).

### Accession numbers

NtCCD4-S (XM_016586431.1; LOC107767418), NtCCD4-T1 (NM_001325517.1; LOC107791323), NtCCD4-T2 (NM_001326122.1; LOC107823578); their nucleotide sequences are 100% identical to those of the cDNA of Tsukuba 1. *Nicotiana sylvestris* CCD4 (XM_009780426.1), *N. tomentosiformis* CCD4-1 (XM_009625204.3) and CCD4-2 (XM_009612833.3).

### Phylogenetic analysis

Neighbor-joining tree with bootstrap resampling was generated by MEGA X^30^. The tree was constructed using full-length amino acid sequences.

### Generation of RNAi transgenic plants

Construction of RNAi cassettes and plant transformation were performed as described previously^22^. Briefly, a coding region of 323 bp that has high identity among *NtCCD4-S*, *-T1* and *-T2* was amplified from a cDNA clone of the *NtCCD4-T2* gene using a set of primers (Supplementary Table S3). The product was subcloned into pENTR/D-TOPO (Thermo Fisher Scientific Waltham, MA, USA). After the sequence was verified, the insert was cloned into an RNAi vector, pSP231, which was derived from pHELLSGATE12^31^ by using a Gateway cloning system (Thermo Fisher Scientific). Transformation of Tsukuba 1 was mediated by *Agrobacterium* (strain LBA4404). Kanamycin was used to select transformed clones. T1 progenies were used for phenotypic analysis.

### NtCCD4 mutants

A library for *N. tabacum* cultivar Tsukuba 1 mutated with ethyl methanesulfonate^23^ was screened as described by Takakura et al.^22^ Briefly, Seeds were immersed in either a 0.6% or 0.8% (w/v) EMS solution for 16 h, then rigorously washed with water before grown in a greenhouse. We collected an equal amount of leaf tissue from eight individual M2 plants which were reproduced from each of the 1974 M1 plants after the mutagenesis. DNA was extracted to create bulked DNA that represented all the induced mutations. Genotypes were determined using PCR and followed by sanger sequencing using the primers listed in Supplementary Table S3. The triple and double mutant lines were isolated from progeny of a hybrid strain that was obtained by crossing the single mutants.

### Carotenoid analysis

Quantitative carotenoid analysis was conducted by Japan Food Research Laboratories (Tokyo, Japan). Total carotenoids were quantified by absorption photometry (absorption coefficient of lutein: E1% 1 cm = 2550, absorption wavelength: 455 nm, solvent: ethanol). Each carotenoid was quantified by HPLC. For semi-quantitative carotenoid analysis, freeze-dried or flue-cured leaves (0.1 g dry weight per analysis) were ground and extracted by shaking at 25 °C with 5.0 mL of acetone/ethanol (1:1, v/v) containing 0.1% 2,6-di-tert-butylhydroxybenzene as an antioxidant. *Trans-*retinol (50 µL) dissolved in the extraction solution (100 µg/mL) was added to each sample as an internal standard. The resultant products were filtered through a 0.45-µm PTFE membrane filter and were analyzed by LC-SRM-MS Agilent 6470 Triple Quad LC/MS equipped with a photodiode array detector (Agilent, Tokyo, Japan). The chromatograms were monitored at 450 nm for carotenoids and at 325 nm for *trans-*retinol. Selected reaction monitoring transitions of each compound used for identification are presented in Supplementary Table S4.

### Semi-quantitative apocarotenoid analysis

Flue-cured leaves (0.1 g dry weight per analysis) were ground and extracted by shaking at 70 °C with 6.0 mL of methanol containing 1,3-dimethoxybenzene (5 µg/mL), added to each sample as an internal standard. The resultant products were filtered through a 0.45-µm PTFE membrane filter and were analyzed by GC-MS 5977A MSD (Agilent) with selected ion monitoring (SIM) mode. SIM parameters (identification and semi-quantification ions) of each compound are presented in Supplementary Table S5. Megastigmatrienone isomers were cited from Slaghenaufi et al.^29^.

### Leaf color analysis

Minolta CR-10 portable colorimeter (Konica Minolta, Tokyo, Japan) with CIEL*a*b* color system was used to measure leaf color. Adaxial color of mature leaves of 94-day-old plants (8 days after topping) grown in the greenhouse was analyzed. Data are the mean of 18 points of three leaves (six points per leaf detached from each individual plant) ± SEM. Adaxial color of flue-cured leaves obtained in the field experiment was analyzed. Data are the mean of 24 points of four leaves (six points per leaf from each individual test area) ± SEM.

### Real-time quantitative RT-PCR

Total leaf RNA was isolated using the RNeasy extraction kit (Qiagen, Valencia, CA, USA). cDNA was synthesized using the PrimeScript RT reagent kit with gDNA Eraser (Takara Bio, Ohtsu, Japan). The quantitative PCR analysis was performed using the TaqMan Fast Advanced Master Mix (Thermo Fisher Scientific) and the StepOnePlus system (Thermo Fisher Scientific). The primers and probes are listed in Supplementary Table S3. The expression levels were normalized to that of the *N. tabacum* *elongation factor-1α* gene (GenBank reference: AF120093) and were calculated using the 2^-ΔΔCt^ method.

### Research involving plants

The flue-cured tobacco (*N. tabacum*) cultivar Tsukuba 1 used in this study was developed at Japan Tobacco Inc. in 1980. This study complied with institutional and national guidelines for experimental research involving plants.

### Supplementary Information

Supplementary Information 1.

Supplementary Information 2.

## Data Availability

All data generated or analyzed during this study are included in the articles and its supplementary information files. The datasets generated and/or analyzed during the current study are available from the corresponding author on reasonable request.

## References

[1] Ruiz-Sola MA, Rodriguez-Concepcion M (2012). Carotenoid biosynthesis in Arabidopsis: A colorful pathway. Arabidopsis Book.

[2] Nisar N, Li L, Lu S, Khin NC, Pogson BJ (2015). Carotenoid metabolism in plants. Mol. Plant.

[3] Sun T (2018). Carotenoid metabolism in plants: The role of plastids. Mol. Plant.

[4] Hou X, Rivers J, León P, McQuinn RP, Pogson BJ (2016). Synthesis and function of apocarotenoid signals in plants. Trends Plant Sci..

[5] Fujimori T, Kasuga R, Matsushita H, Kaneko H, Noguchi M (1976). Neutral aroma constituents in burley tobacco. Agric. Biol. Chem..

[6] Mookherjee BD, Wilson RA (1990). Tobacco constituents—Their importance in flavor and fragrance chemistry. Perfum. Flavorist.

[7] Yokoi M, Shimoda M (2017). Extraction of volatile flavor compounds from tobacco leaf through a low-density polyethylene membrane. J. Chromatogr. Sci..

[8] Popova V (2019). Carotenoid-related volatile compounds of tobacco (*Nicotiana tabacum* L.) essential oils. Molecules.

[9] Ito K (2000). Glycosidic fraction of flue-cured tobacco leaves: Its separation and component analysis. Biosci. Biotechnol. Biochem..

[10] Winterhalter, P., & Rouseff R. L. in *Carotenoid-Derived Aroma Compounds* Vol. 802 (eds Winterhalter, P., Rouseff, R. L.) 1, Ch. 1 (2001).

[11] Court WA, Hendel JG (1984). Changes in leaf pigments during sensescence and curing of flue-cured tobacco. Can. J. Plant Sci..

[12] Wu S (2020). Comparative proteomic analysis by iTRAQ reveals that plastid pigment metabolism contributes to leaf color changes in tobacco (*Nicotiana tabacum*) during curing. Int. J. Mol. Sci..

[13] Zhou Q (2019). Carotenoid cleavage dioxygenases: Identification, expression, and evolutionary analysis of this gene family in tobacco. Int. J. Mol. Sci..

[14] Iuchi S (2001). Regulation of drought tolerance by gene manipulation of 9-cis-epoxycarotenoid dioxygenase, a key enzyme in abscisic acid biosynthesis in Arabidopsis. Plant J..

[15] Tan BC, Schwartz SH, Zeevaart JAD, McCarty DR (1997). Genetic control of abscisic acid biosynthesis in maize. Proc. Natl. Acad. Sci..

[16] Alder A (2012). The path from b-carotene to carlactone, a strigolactone-like plant hormone. Science.

[17] Wang JY (2019). The apocarotenoid metabolite zaxinone regulates growth and strigolactone biosynthesis in rice. Nat. Commun..

[18] Ohmiya A (2019). Molecular basis of carotenoid accumulation in horticultural crops. Hortic. J..

[19] Ohmiya A, Kishimoto S, Aida R, Yoshioka S, Sumitomo K (2006). Carotenoid cleavage dioxygenase (CmCCD4a) contributes to white color formation in chrysanthemum petals. Plant Physiol..

[20] Campbell R (2010). The metabolic and developmental roles of carotenoid cleavage dioxygenase4 from potato. Plant Physiol..

[21] Gonzalez-Jorge S (2013). Carotenoid cleavage dioxygenase4 is a negative regulator of beta-carotene content in Arabidopsis seeds. Plant Cell.

[22] Takakura Y, Udagawa H, Shinjo A, Koga K (2018). Mutation of a *Nicotiana tabacum* L. eukaryotic translation-initiation factor gene reduces susceptibility to a resistance-breaking strain of Potato virus Y. Mol. Plant Pathol..

[23] Tajima T, Sato S, Hiyoshi T (2011). Construction of mutant panel in *Nicotiana tabacum* L. Ann. Phytopathol. Soc. Jpn..

[24] Rottet S (2016). Identification of plastoglobules as a site of carotenoid cleavage. Front. Plant Sci..

[25] Cai K (2013). Identification and quantitation of glycosidically bound aroma compounds in three tobacco types by gas chromatography–mass spectrometry. J. Chromatogr. A.

[26] Latari K (2015). Tissue-specific apocarotenoid glycosylation contributes to carotenoid homeostasis in Arabidopsis leaves. Plant Physiol..

[27] Zheng X, Yang Y, Al-Babili S (2021). Exploring the diversity and regulation of apocarotenoid metabolic pathways in plants. Front. Plant Sci..

[28] Ramel F (2012). Carotenoid oxidation products are stress signals that mediate gene responses to singlet oxygen in plants. Proc. Natl. Acad. Sci..

[29] Slaghenaufi D, Perello MC, Marchand S, de Revel G (2016). Quantification of megastigmatrienone, a potential contributor to tobacco aroma in spirits. Food Chem..

[30] Kumar S, Stecher G, Li M, Knyaz C, Tamura K (2018). MEGA X: Molecular evolutionary genetics analysis across computing platforms. Mol. Biol. Evol..

[31] Wesley SV (2001). Construct design for efficient, effective and high-throughput gene silencing in plants. Plant J..

