# Supplementary material for: Multiple loss-of-function mutations of carotenoid cleavage dioxygenase 4 reveal its major role in both carotenoid level and apocarotenoid composition in flue-cured mature tobacco leaves
Source: Sci Rep. 2023 Aug 10;13:12992. doi: 10.1038/s41598-023-39692-4 (PMC10415294; doi:10.1038/s41598-023-39692-4)
Supplement: Supplementary file 1 — Supplementary Information 1. [file 41598_2023_39692_MOESM1_ESM.pptx]

## Slide 1
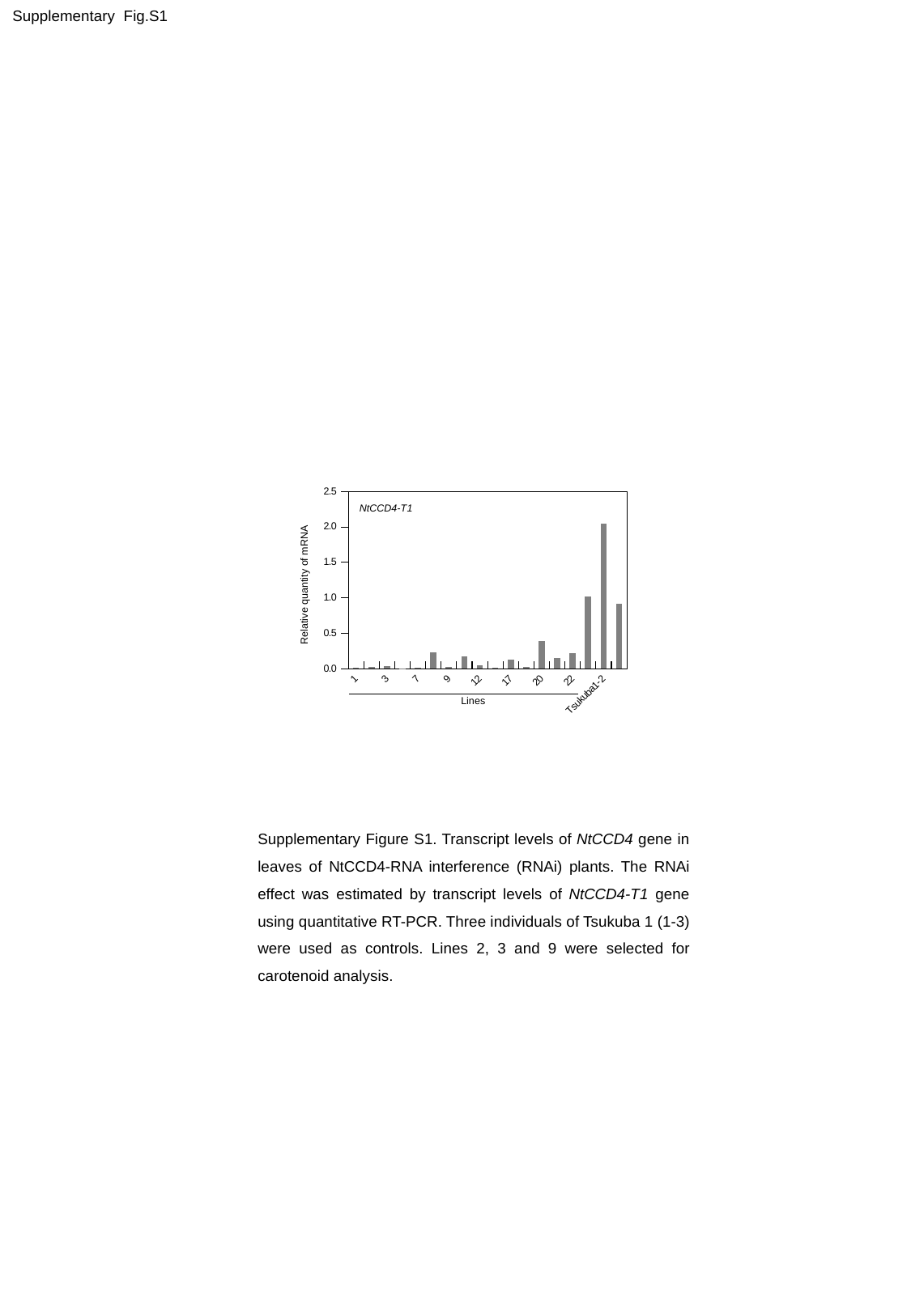

Supplementary Fig.S1
### Chart
| Category | CCD4-1 |
|---|---|
| 1 | 0.018129775300621986 |
| 2 | 0.018920764327049255 |
| 3 | 0.04100722074508667 |
| 5 | 0.006382375489920378 |
| 7 | 0.007216850761324167 |
| 8 | 0.2279033213853836 |
| 9 | 0.025107230991125107 |
| 10 | 0.16925135254859924 |
| 12 | 0.04274662956595421 |
| 15 | 0.007427207659929991 |
| 17 | 0.12696875631809235 |
| 18 | 0.02863740175962448 |
| 20 | 0.39510470628738403 |
| 21 | 0.15472838282585144 |
| 22 | 0.22217021882534027 |
| Tsukuba1-1 | 1.0221120119094849 |
| Tsukuba1-2 | 2.04790997505188 |
| Tsukuba1-3 | 0.9182884097099304 |NtCCD4-T1
Relative quantity of mRNA
Lines
Supplementary Figure S1. Transcript levels of NtCCD4 gene in leaves of NtCCD4-RNA interference (RNAi) plants. The RNAi effect was estimated by transcript levels of NtCCD4-T1 gene using quantitative RT-PCR. Three individuals of Tsukuba 1 (1-3) were used as controls. Lines 2, 3 and 9 were selected for carotenoid analysis.

## Slide 2
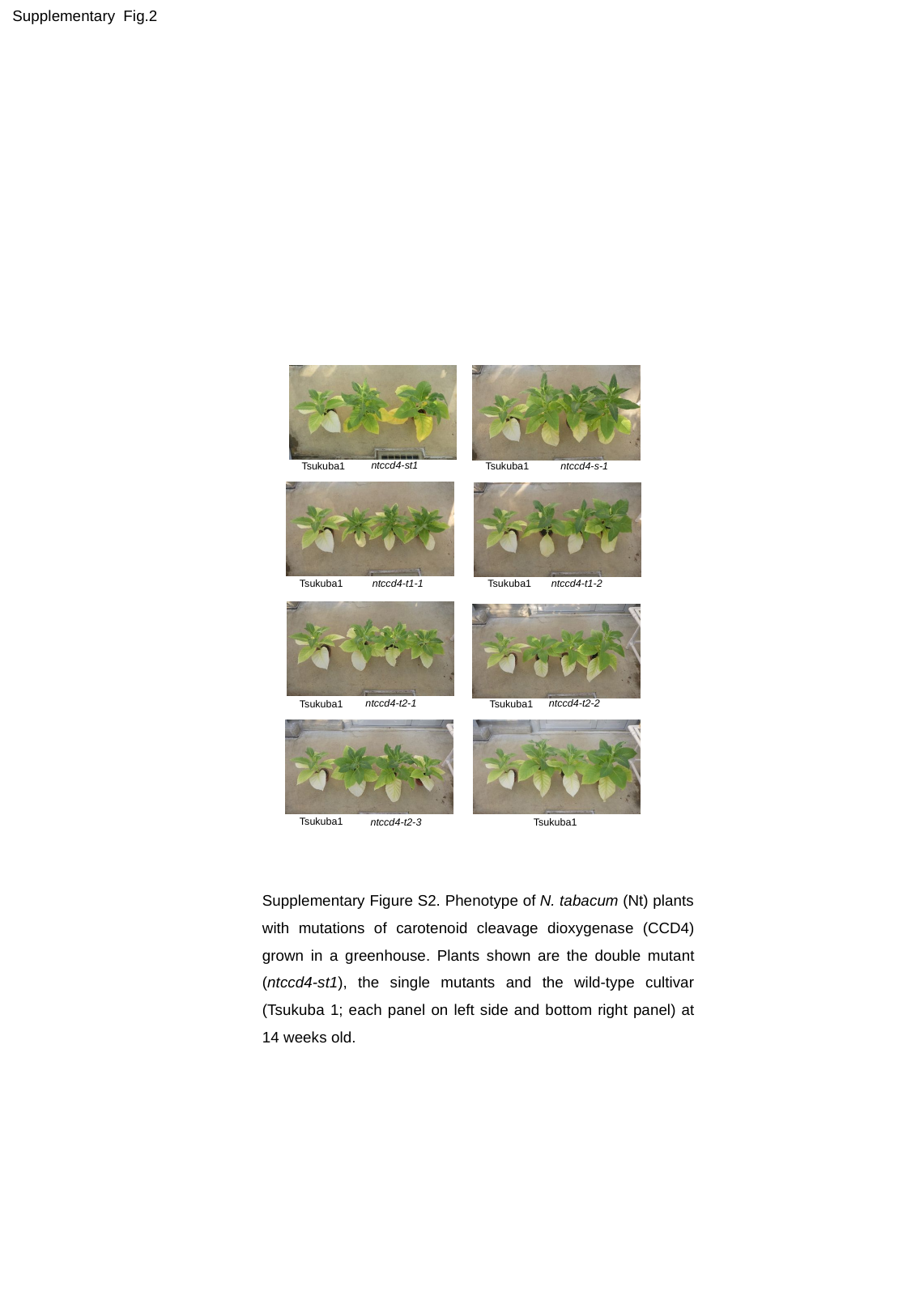

Supplementary Fig.2
ntccd4-st1
ntccd4-s-1
Tsukuba1
Tsukuba1
Tsukuba1
ntccd4-t1-1
ntccd4-t1-2
Tsukuba1
ntccd4-t2-1
ntccd4-t2-2
Tsukuba1
Tsukuba1
Tsukuba1
ntccd4-t2-3
Tsukuba1
Supplementary Figure S2. Phenotype of N. tabacum (Nt) plants with mutations of carotenoid cleavage dioxygenase (CCD4) grown in a greenhouse. Plants shown are the double mutant (ntccd4-st1), the single mutants and the wild-type cultivar (Tsukuba 1; each panel on left side and bottom right panel) at 14 weeks old.

## Slide 3
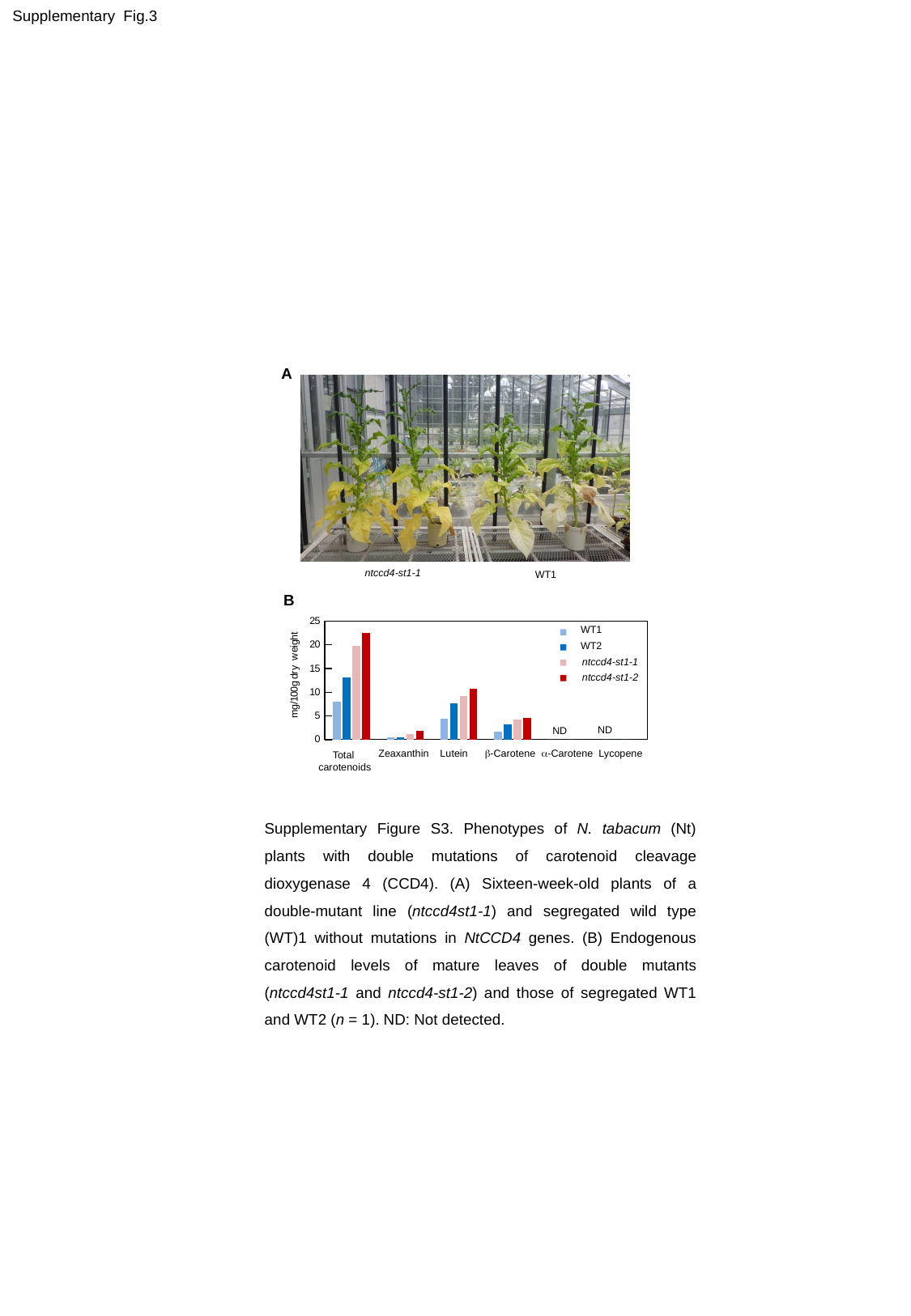

Supplementary Fig.3
A
ntccd4-st1-1
WT1
B
### Chart
| Category | WT1 | WT2 | CCD4-st1-1 | CCD4-st1-2 |
|---|---|---|---|---|
| Total　carotenoids | 7.97 | 13.1 | 19.7 | 22.5 |
| zeaxanthin | 0.43 | 0.45 | 1.08 | 1.8 |
| lutein | 4.43 | 7.6 | 9.15 | 10.8 |
| b-carotene | 1.6 | 3.26 | 4.26 | 4.59 |
| a-carotene | 0.0 | 0.0 | 0.0 | 0.0 |
| lycopene | 0.0 | 0.0 | 0.0 | 0.0 |WT1
WT2
ntccd4-st1-1
ntccd4-st1-2
ND
ND
Zeaxanthin Lutein b-Carotene a-Carotene Lycopene
Total
carotenoids
Supplementary Figure S3. Phenotypes of N. tabacum (Nt) plants with double mutations of carotenoid cleavage dioxygenase 4 (CCD4). (A) Sixteen-week-old plants of a double-mutant line (ntccd4st1-1) and segregated wild type (WT)1 without mutations in NtCCD4 genes. (B) Endogenous carotenoid levels of mature leaves of double mutants (ntccd4st1-1 and ntccd4-st1-2) and those of segregated WT1 and WT2 (n = 1). ND: Not detected.
